# Assessment of the Effect of Thermoforming Process and Simulated Aging on the Mechanical Properties of Clear Aligner Material

**DOI:** 10.7759/cureus.64933

**Published:** 2024-07-19

**Authors:** Manjiri Bhate, Shweta Nagesh

**Affiliations:** 1 Orthodontics and Dentofacial Orthopedics, Saveetha Dental College and Hospitals, Saveetha Institute of Medical and Technical Sciences, Saveetha University, Chennai, IND

**Keywords:** dental biomaterial, material properties, thermocycling, theromoforming, orthodontics, clear aligner

## Abstract

Background

Choosing the optimal aligner material on the market is crucial to ensure constant forces for tooth displacement. Processes like manufacturing and intraoral usage can result in the degradation of certain properties, which can affect the overall efficacy of treatment.

Objective

The objective of the study is to compare the surface roughness and flexural modulus of two aligner materials following the processes of thermoforming and aging.

Materials and methods

Two groups of 12 samples each were tested: Group 1 consisted of polyethylene terephthalate glycol (PET-G) and Group 2 of zendura-polyurethane (PU). The groups were tested at three time points: T0 - pre-thermoformed; T1 - after thermoforming; T2 - after thermoforming and aging. The surface roughness and the flexural modulus were evaluated. One-way ANOVA followed by a Bonferroni post hoc test was conducted to compare the changes within each group across the three times. An independent t-test was done to compare the values between the two groups at each time point. The statistical tests were performed using SPSS software version 26 (IBM Corp., Armonk, NY, USA). P-values >0.05 were considered statistically significant.

Results

There was a significant change in the surface roughness post-aging in Group 2 (p=0.03) and flexural strength within Group 1 (p=0.031) and Group 2 (p=0.06) across the three time points. Comparing the changes within the three time points in Group 1, significant changes were observed between T0-T1 (p=0.045) and T0-T2 (p=0.07). In Group 2, significant changes were observed between T0-T2 (p=0.012). Comparing the flexural strength between the two groups, significant differences were observed at T0 (p=0.012) and T1 (p=0.001).

Conclusion

The aging process affected the surface roughness in Zendura (PU). The thermoforming and aging process resulted in reduced flexural strength in both Zendura (PU) and Duran groups (PET-G).

## Introduction

The development of clear aligners has undergone significant advancements over the last ten years due to the growing emphasis on esthetics [1]. Compared to fixed appliances, clear aligners offer several advantages, including improved esthetics [2] and better periodontal health [3,4]. The common method for manufacturing aligners involves the use of 3D printing technology to fabricate 3D models that depict staged tooth motions. These models are then used to thermoform aligner sheets, resulting in the production of aligners [5,6]. The efficacy of thermoformed aligner treatment is mostly contingent upon the characteristics of the plastic foil employed, the process by which it is transformed into an aligner, and the precision of dental model printing [7]. The manufacturing process can potentially cause changes in the properties of the aligners produced, hence necessitating careful evaluation to determine the resultant forces exerted on teeth [8]. Multiple investigations have documented that the mechanical and physical characteristics of orthodontic aligner materials can undergo alterations subsequent to thermoforming, cyclic mechanical loading, thermocycling processes, and clinical utilization [9]. Given the increasing number of companies producing aligner materials and the emergence of in-office aligners, it is crucial to have knowledge about the characteristics of different thermoforming materials available on the market and how these properties may be altered during the manufacturing process and clinical application [10].

Clear aligners are made from thermoplastic resin polymers, including polyurethane (PU), polyethylene terephthalate (PET), polyethylene terephthalate glycol (PET-G), and polyvinyl chloride (PVC). Various aligner brands use different materials [11]. These polymers are susceptible to alterations when exposed to factors such as elevated temperatures, humidity, sustained mechanical stress, and the presence of saliva within the oral cavity [12]. Surface roughness and flexural strength are two important properties of aligner materials with high clinical significance. Despite documented evidence indicating that aligners exhibit reduced plaque retention and a lower incidence of gingival inflammation compared to fixed appliances, there is also evidence suggesting that significant alterations in supragingival microbiology can still occur with both fixed and removable appliances. The bio-adhesion phenomena and the subsequent formation of pellicle and plaque are essentially determined by the surface morphology, chemistry, and charge of the aligner material [13]. Furthermore, it is imperative that characteristics such as tensile strength, compressive strength, and flexural strength remain consistent during the duration of therapy. Specifically, if the properties of the aligner are altered after fabrication and during usage, the orthodontist's ability to regulate the force exerted on the teeth and their positioning may be compromised [14]. Hence, it is crucial to evaluate the alterations in mechanical properties subsequent to the thermoforming process. A study by Dalaie K et al. [12] assessed the mechanical and thermal properties after the thermoforming process and aging in Duran and Erkodur aligner materials and found that the thermoforming process had a more significant effect on the thermomechanical properties of the aligner materials. Though many studies have examined the effects of thermoforming and aging on material properties, there remains a lack of literature specifically evaluating surface roughness and flexural modulus in two commonly used aligner materials. Hence, the present in-vitro study aims to compare and evaluate the surface roughness and flexural modulus of two aligner materials (PET-G and PU) after thermoforming and aging.

## Materials and methods

The present in-vitro study was commenced after approval from the institutional review board (SRB/SDC/ORTHO-2206/23/063). Ethical approval and patient consent were not applicable for this study. The sample size was calculated using results obtained in the study by Dalaie K et al. [12] using G*Power software (version 3.1). Considering an α error equivalent to 0.05 and the power of the study equivalent to 0.95, the sample size was determined to be 24, divided into two groups of 12 samples each. Group 1 comprised Duran material (Scheu-Dental GmbH, Iserlohn, Germany) with a thickness of 0.75 mm - PET-G, and Group 2 comprised Zendura material (Bay Materials LLC, Fremont, CA, USA) with a thickness of 0.75 mm - thermoplastic PU.

**Figure 1:**
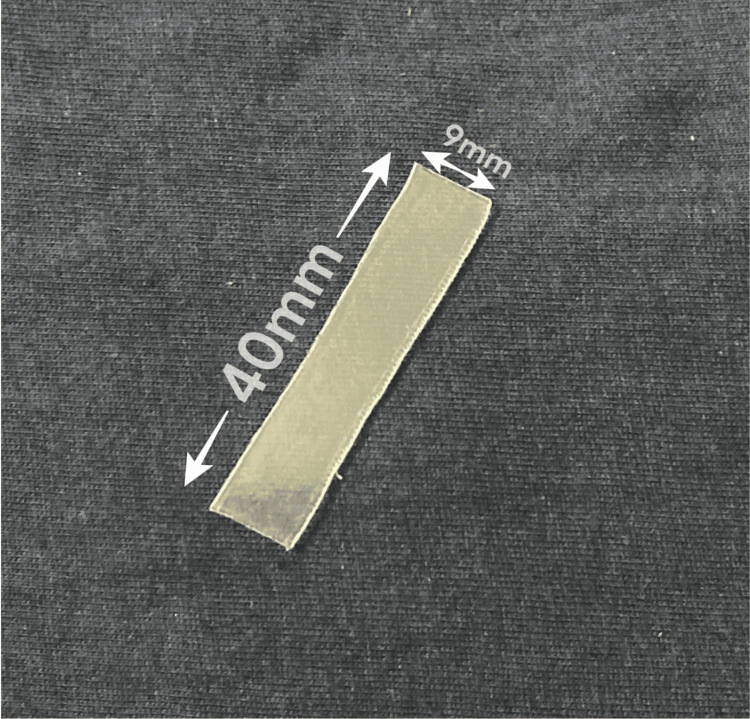
Aligner material sample prepared in standard dimensions.

The samples of the two groups were measured at three different time points: T0 - pre-thermoformed; T1 - after thermoforming; T2- after thermoforming and aging. The aligner material samples were prepared and standardized based on the material testing parameters suggested in a previous study [15] for valid and comparable results. Samples from the six groups were cut to a standard dimension of 40x9mm (Figure 1).

Four samples in each group were cut to these dimensions prior to thermoforming. All other samples were thermoformed on a flat metal plate and then cut to the specified dimensions. Among the thermoformed samples, four samples in each group underwent testing, and the remaining samples were subjected to simulated intraoral aging and were then tested. The thermoforming process was conducted using the Biostar Thermoforming Machine (Scheu-Dental GmbH, Iserlohn, Germany). The sheets were not subjected to thermoforming over a dental cast due to the experimental requirements necessitating the use of flat and uniformly sized samples. Therefore, the process of thermoforming was conducted on a flat steel surface. Subsequently, the sheet was divided into the aforementioned dimensions.

A total of four samples per group underwent simulated aging subsequent to the thermoforming process. The process of aging was simulated by subjecting the samples to thermocycling, which replicates the temperature fluctuations experienced in the oral environment. Following the thermoforming process, the samples were immersed in distilled water at a temperature of 37°C for a duration of 24 hours. Subsequently, thermocycling was performed. Given that each aligner is typically worn for a minimum duration of two weeks, it was determined that subjecting the aligner to 14 thermal cycles every day, resulting in a total of 200 cycles over the course of two weeks, would effectively replicate the conditions of the oral environment. This duration for thermocycling was based on the typical time period during which one standard aligner is typically worn continuously [12].

Test for surface roughness

Surface roughness for all the samples in all groups was tested using a surface profilometer (Mitutoyo Surface Profilometer SJ 310, Romania). All samples were tested thrice, and the average value for each sample was considered for statistical analysis.

Test for flexural modulus

**Figure 2:**
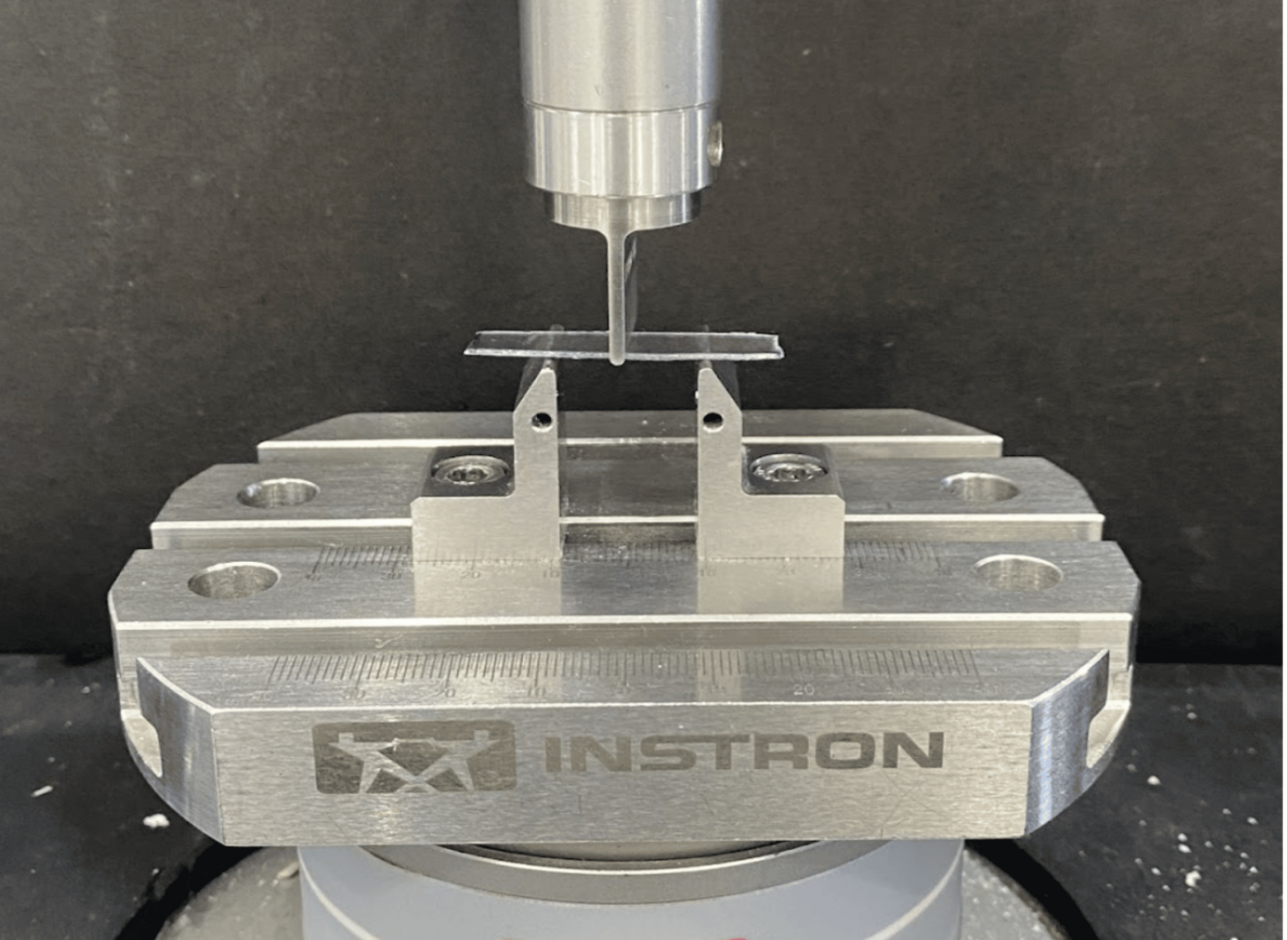
Flexural modulus of the samples assessed using three-point bend test in a universal testing machine.

The flexural modulus for all samples was assessed using a three-point bending test on an Instron E-3000 Universal Testing Machine (Instron Corp., MA, USA). The methodology was based on the study by Elkholy F et al. [15]. Each sample was loaded until it broke at a cross-head speed of 1 mm/min (Figure 2).

Statistical analysis

The statistical analysis was performed using SPSS software version 26 (IBM Corp., Armonk, NY, USA). Normality of the data was assessed using the Shapiro-Wilk test. The differences in surface roughness and flexural strength between the two groups at three timepoints were assessed using repeated measures ANOVA, followed by intergroup comparison using the Bonferroni test. Parameters were compared at each time point between Groups 1 and 2 using an independent t-test. The significance level was set at 0.05.

## Results

The study assessed the change in surface roughness and flexural modulus after thermoforming and simulated intraoral aging. The data from the experiments were tabulated and subjected to statistical analysis. Normality assessment was conducted using the Shapiro-Wilk test, which found the data to be normally distributed, and parametric tests were utilized for assessment of statistical significance. The results are elaborated below.

Surface roughness

The mean surface roughness in Groups 1 and 2 at T0 was 0.07±0.01 µm and 0.06±0.03 µm, respectively. At T1, the mean surface roughness in Groups 1 and 2 was 0.09±0.04 µm and 0.07±0.01 µm, respectively. At T2, the mean surface roughness in Group 1 was 0.06±0.08 µm, and in Group 2, it was 0.25±0.11 µm. Comparing the mean surface roughness of Group 1 across three time points (T0-T2), no statistically significant differences were found. However, a significant statistical difference in surface roughness was observed among the three time points in Group 2 (p=0.031). The values have been tabulated in Table 1.

**Table 1:** Comparison of surface roughness and flexural strength in Groups 1 and 2. Mean and SD of the surface roughness value (Ra) and the flexural strength in Groups 1 and 2 at three time points: T0 - Before thermoforming and aging; T1 - After thermoforming; T2 - After thermoforming and aging. Repeated measures ANOVA was conducted to compare the changes in the values across the time points. A p-value of less than or equal to 0.05 is considered statistically significant.

Group 1				
Surface roughness (µm)			Flexural strength (N)	
Time points	Mean and SD	P-value	Mean and SD	P-value
T0	0.07±0.01	0.562	1.93±0.18	0.03*
T1	0.09±0.04		1.77±0.13	
T2	0.06±0.08		1.39±0.06	
Group 2				
Surface roughness (µm)			Flexural strength (N)	
Time points	Mean and SD	P-value	Mean and SD	P-value
T0	0.06±0.03	0.031*	1.28±0.16	0.06*
T1	0.07±0.01		1.3±0.46	
T2	0.25±0.11		0.51±0.2	

Pairwise comparisons were conducted to assess the change in surface roughness between the time points in both groups. No significant differences were found in Group 1. However, in Group 2, there was a significant increase in surface roughness between T1 and T2, with a p-value of 0.013 (Table 2).

**Table 2:** Pairwise comparison using Bonferroni test. P-value of less than or equal to 0.05 is considered statistically significant.

Group 1			
Surface roughness (µm)		Flexural strength (N)	
Groups compared	P-value	Groups compared	P-value
T0-T1	0.956	T0-T1	0.045*
T0-T2	0.639	T0-T2	0.007*
T1-T2	0.398	T1-T2	0.815
Group 2			
Surface roughness ($\mu$m)		Flexural strength (N)	
Groups compared	P-value	Groups compared	P-value
T0-T1	0.479	T0-T1	1
T0-T2	0.077	T0-T2	0.012*
T1-T2	0.013*	T1-T2	0.127

When comparing the mean surface roughness using an independent t-test between Groups 1 and 2 at each time point, no statistically significant differences were observed (Table 3).

**Table 3:** Comparison between Group 1 and Group 2. Comparison between Group 1 and Group 2 at each time point was done using independent t-test. P-value of less than or equal to 0.05 is considered statistically significant.

Surface roughness (µm) (Group 1 vs. Group 2)		Flexural strength (N) (Group 1 vs. Group 2)	
Time point	P-value	Time point	P-value
T0	0.942	T0	0.012*
T1	0.08	T1	0.001*
T2	0.059	T2	0.526

Flexural strength

The mean and standard deviation of the flexural strength for Group 1 at three different time points (T0-T2) are listed in Table 1. Duran material at T0 showed the highest flexural strength (1.93±0.18N). A substantial decrease in flexural strength was detected when comparing the data at all three time points (p=0.03). Statistically significant differences were observed between T0-T1 (p=0.045) and T0-T2 (p=0.007) using pairwise comparison with the Bonferroni test (Table 2). The mean and standard deviation of the flexural strength for Group 2 at three different time points (T0-T2) are also listed in Table 1. An evident reduction in flexural strength was found at the three time points, with statistical significance (p=0.06). Pairwise analysis revealed statistically significant changes between T0 and T2 time points (p=0.012) as shown in Table 2.

An independent t-test revealed that Group 2 had a reduction in flexural strength relative to Group 1 at both T0 (p=0.012) and T1 (p=0.001). The disparities were statistically significant (Table 3).

## Discussion

The thermal changes that aligner materials undergo during thermoforming can lead to intricate structural changes, which may affect the material's mechanical properties, including transparency, Young's modulus, thickness, surface roughness, and flexural modulus [16]. Hygrothermal aging is a technique extensively employed in dental research to simulate intraoral aging. It involves the consecutive immersion of materials in purified water at varying temperatures under laboratory conditions [16]. The present study assessed the effect of these processes on the surface roughness and flexural modulus of two commercially available aligner materials (PET-G and PU). It found that the PET-G (Duran) material (Group 1) did not exhibit any significant alterations in surface roughness following thermoforming and aging. In contrast, the PU (Zendura) materials (Group 2) showed an increase in surface roughness after undergoing thermoforming and aging. However, upon comparing the two groups, no significant change was noticed. Albilali AT et al. [17] conducted a study that evaluated the alterations in several mechanical characteristics of thermoformable materials after undergoing thermoforming and aging. The study determined that there was no notable alteration in surface roughness after the thermoforming process. However, after undergoing both thermoforming and aging, Zendura exhibited the greatest degree of surface roughness. Although the surface roughness value achieved in the current investigation was lower than that of the study described previously, the surface roughness of the PU material increased with aging, similar to the prior study.

In the study by Šimunović L et al. [18], the surface roughness and flexural modulus of four different types of thermoplastic aligner materials, namely Zendura, Zendura FLX, Duran, and CA Pro, were evaluated after undergoing thermoforming and aging. The surface roughness value (Ra) did not exhibit any statistically significant differences among the materials, which is consistent with the findings of the current investigation. In a study by Suter F et al. [13], various commercially available brands of thermoformable materials based on PET-G were assessed for surface roughness and wettability following the thermoforming process alone. The study did not find any significant changes in surface roughness, similar to the present study. Staderini E et al. [19] conducted a study where they observed a notable increase in the surface roughness of PET-G material after thermoforming, as determined by atomic force microscopy. The findings of the current investigation were incongruent with those of the prior study. This can be related to the different approach used to evaluate the roughness of the surface. The current investigation employed a stylus profilometer, a device that offers a quantitative assessment of surface roughness. However, advanced imaging with atomic force microscopy may offer a qualitative and very accurate evaluation of surface topography [20]. The current investigation assessed the mean roughness (Ra) of the materials. It established that the average roughness (Ra) of dental materials must be less than 0.2 µm to avoid the accumulation of plaque and bacteria. Increased surface roughness results in a progressive buildup of plaque over time. Research indicates that if the roughness value of an intraoral hard surface surpasses 0.5 µm, it might induce discomfort and be sensed by the tongue [20]. Both groups tested in the present study produced surface roughness values less than 0.2 µm, except for the PU (Group 2) material after thermoforming and aging (Ra = 0.25±0.11 µm).

The current study observed a substantial decrease in the flexural strength of both PET-G (Group 1) and PU (Group 2) materials following thermoforming and aging. It is worth mentioning that Group 2 exhibited considerably reduced flexural strength compared to Group 1, both before and after thermoforming. However, after the aging process, no notable disparity was observed between the two groups. In the study by Albilali AT et al. [17], no significant differences in the flexural modulus between Zendura and Duran materials were observed post thermoforming and aging, unlike the findings of the present study. Additionally, that study found an increase in the flexural modulus post-thermoforming, unlike the findings of the present study. In another investigation conducted by Šimunović L et al. [18], it was discovered that the initial flexural modulus in Duran was substantially higher than in Zendura, which aligns with the results of the current study. After undergoing thermoforming and aging, the Duran material exhibited an increase in flexural modulus, which is contrary to the findings of the current investigation. On the other hand, the flexural modulus of Zendura declined. In the study by Dalaie K et al. [12], flexural strengths of PET-G (Duran) and Copolyester material (Erkodur) were compared post thermoforming and aging. The study observed that both materials had a notable decrease in flexural modulus after undergoing thermoforming and thermocycling processes, similar to the present investigation. The study by Ryu JH et al. [21] assessed the changes in mechanical properties of the aligner materials with varying thickness after the thermoforming process. It was observed that the flexural force decreased after thermoforming in 0.75 mm of Duran (PET-G) material, similar to the present study. However, changes after aging were not evaluated. Though both groups investigated in the present study showed changes in flexural strength post thermoforming and aging, Duran showed the most change post thermoforming, despite having the highest flexural strength at T0. This can be explained by the increased vulnerability of PET-G polymers to high temperatures between 100 and 130 °C, which can lead to warping, bending, and deformation [18].

Based on the results of the study, PET-G material did not exhibit significant changes in surface roughness by either thermoforming or aging. However, the aging process has a greater influence on the surface roughness of PU material than the thermoforming process. Also, the aging process has a considerable effect on the flexural modulus compared to the thermoforming process. Therefore, materials that have the lowest susceptibility to changes caused by these processes should be used. Furthermore, due to the significant impact of intraoral aging on the properties of aligners, it is not advisable to wear a single aligner for an extended period. This must be considered during aligner planning.

An important limitation of the present study is the in-vitro study design. The study utilized thermocycling to simulate intra-oral aging, which is considered an appropriate technique for expediting the artificial aging of the samples compared to other methods like static immersion techniques. The clinical efficacy can be estimated by reproducing the temperature of the oral environment [22]. Even though the aging process was simulated using thermocycling, various factors like plaque, bacterial activity, and occlusal loading can alter the mechanical properties of the aligner materials, which has not been taken into consideration in the present study. The surface roughness was assessed using a contact profilometer in this study. The contact profilometer utilizes a highly sensitive stylus to trace and define the surface features [23]. However, more advanced techniques like atomic force microscopy can detect the changes with much more detail. In biomechanical assessment of aligners using three-point bending, it is typical to employ flat specimens [15]. The omission of the intricate 3D morphology of aligners utilized in clinical therapy is another limitation. This can imply an absence of direct clinical significance in the magnitudes of the forces evaluated. Given the use of newer materials, such as thermoformed and direct printed aligners, in clear aligner therapy, it is crucial to conduct additional research to gain a better understanding of how these materials interact with the oral environment. Further research is necessary to assess the impact of different manufacturing processes and intraoral conditions on the characteristics and behavior of these materials.

## Conclusions

From the study, it can be inferred that thermoforming and aging did not result in any clinically or statistically significant alterations in the surface roughness of the Duran material. Although Zendura showed a statistically significant change in surface roughness after the aging process, the level of change was not clinically significant enough to promote bacterial accumulation. Both Zendura and Duran groups showed a substantial decrease in flexural strength due to the thermoforming and aging process. The process of simulated intraoral aging affected the flexural strength more than the surface roughness in both groups.
